# Bacterial peptidoglycan enhances sickness behaviour induced by bacterial lipopolysaccharide

**DOI:** 10.1186/1471-2210-11-S2-A14

**Published:** 2011-09-05

**Authors:** Aitak Farzi, Evelin Painsipp, Peter Holzer

**Affiliations:** 1 Institute of Experimental and Clinical Pharmacology, Medical University of Graz, 8010 Graz, Austria

## Background

Lipopolysaccharide (LPS) and peptidoglycan are microbial products recognized by Toll-like receptor-4 (TLR4) and nucleotide-binding oligomerization domain 1 (NOD1) and NOD2, respectively. LPS has been found to cause behavioural alterations indicative of sickness and depressed mood. The effect of peptidoglycan on exploratory and affective behaviour has not yet been explored, although it has been reported that it promotes sleep and anorexia. Since NOD1 and NOD2 are activated by two different peptidoglycan components, MurNAc-L-Ala-γ-D-Glu-meso-diaminopimelic acid (M-TriDAP) and muramyl dipeptide (MDP), respectively, the effects of both compounds, alone and in combination with LPS, on exploratory and affective behaviour were investigated.

## Methods

Female C57BL/6 mice received intraperitoneal injections of M-TriDAP (100 µg/mouse), MDP (10 mg/kg) or sterile saline (0.9% NaCl) and additional injections of LPS (0.83 mg/kg) or sterile saline 4 hours after the first injection. The weight of the animals was monitored throughout the study. Exploratory and anxiety-like behaviour was evaluated with the elevated plus-maze test (EPM) 1 day after treatment, while depression-related (stress coping) behaviour was assessed with the forced swim test (FST) 1 week after treatment.

## Results

Mice receiving sterile saline plus LPS, M-TriDAP plus LPS and MDP plus LPS, respectively, lost body weight by 12% during the first day after treatment, which was reversed 1 week after treatment. When given alone, M-TriDAP and MDP failed to change the body weight. Likewise, M-TriDAP and MDP alone did not alter behaviour on the EPM as tested 1 day after treatment. LPS alone decreased exploratory behaviour in the EPM as displayed by a nonsignificant reduction of the total travelling distance and number of arm entries. When given in combination with LPS, especially MDP enhanced the effect of LPS. Specifically, the decrease of the total travelling distance and the total number of arm entries reached statistical significance. When tested in the FST, the animals tended to exhibit increased immobility and decreased activity (swimming and climbing), an effect that was again most pronounced in the MDP plus LPS group. However, this effect was not statistically significant.

## Conclusions

The loss of body weight and decrease in exploratory behaviour following administration of bacterial immune stimulants reflect sickness behaviour associated with infection. The present results reveal that MDP, a NOD2 agonist, enhances the sickness behaviour caused by LPS. This observation indicates that the brain response to peripheral immune activation depends on both TLR4 and NOD2.

